# Effects of Inorganic Zn and Cu Supplementation on Gut Health in Broiler Chickens Challenged With *Eimeria* spp.

**DOI:** 10.3389/fvets.2020.00230

**Published:** 2020-04-28

**Authors:** Tatiane Souza dos Santos, Po-Yun Teng, Sudhir Yadav, Fernanda Lima de Souza Castro, Rebecca Lizabeth Gould, Steven Wesley Craig, Chongxiao Chen, Alberta Lorraine Fuller, Robert Pazdro, José Roberto Sartori, Woo Kyun Kim

**Affiliations:** ^1^Department of Breeding and Animal Nutrition, School of Veterinary Medicine and Animal Science, São Paulo State University (UNESP), Botucatu, Brazil; ^2^Department of Poultry Science, University of Georgia (UGA), Athens, GA, United States; ^3^Department of Foods and Nutrition, University of Georgia (UGA), Athens, GA, United States

**Keywords:** broilers, coccidiosis, copper, gut health, zinc

## Abstract

An experiment was conducted to evaluate the effect of different levels of inorganic copper and zinc on growth performance, intestinal permeability, intestinal lesion scores, oocyst shedding, antioxidant properties and bone quality in broilers challenged with *Eimeria* spp. A total of 360 d-old male Cobb broiler chickens were housed in floor cages for 12 days at the Poultry Research Center. At 12 days of age, birds were placed in grower Petersime batteries and distributed in a completely randomized design with 10 birds per cage, six replicates per treatment, and six treatments. There were six corn-soybean meal-based dietary treatments: non-challenged control (NC), challenged control (CC), 100 ppm Cu (100 Cu), 150 ppm Cu (150 Cu), 80 ppm Zn (80 Zn), and 100 ppm Zn (100 Zn). Broilers received the treatment diets for 9 days (12–20d). Birds, except NC, were challenged with *Eimeria maxima* (50,000 oocysts/bird), *Eimeria tenella* (50,000 oocysts/bird), and *Eimeria acervulina* (250,000 oocysts/bird) on 14d. On 20d, the growth performance was recorded, and one bird/cage was used for analysis of intestinal permeability, antioxidant properties and bone quality. Lesion score was recorded at 20 days of age in eight birds/cage. The means were subjected to ANOVA and, when significant, compared by Duncan's test. Intestinal permeability was significantly improved when birds received the 100 Zn diet (*P* < 0.05). In addition, lesion scores on duodenum were reduced when broilers received diets 150 Cu as compared to CC diet (*P* < 0.05). However, growth performance was not positively influenced by inclusion of inorganic minerals as compared to the NC diet. Furthermore, activity of superoxide dismutase and bone quality were not affected, whereas glutathione status was improved with mineral supplementation in all groups. This study showed that Cu and Zn supplementation improves intestinal integrity during the *Eimeria* spp. infection, suggesting that Cu and Zn supplementation would be a potential strategy to reduce detrimental effects of *Eimeria* infection in broilers.

## Introduction

Coccidiosis is a disease caused by *Eimeria* spp. and causes a negative economic impact on the poultry industry, inducing global losses over 2.4 billion dollars annually (1). Specific *Eimeria* spp. identified in poultry includes *E. acervulina, E. brunetti, E. maxima, E. necatrix, E. praecox, E. mitis, E. tenella, E. mivatti*, and *E. hagani* (2). *Eimeria* spp. colonize in the intestine from duodenum to ceca, leading to inflammation, hemorrhaging, and diarrhea. Damaged intestine caused by coccidiosis also results in increase of gut permeability, reduction of digestibility, poor growth performance, and high mortality in severe infection (3).

Copper (Cu) is an essential mineral in maintaining efficient growth performance and body metabolisms. Cu plays an important role in the electrons transfer in the enzymatic activity of oxidation-reduction (4) during formation of hemoglobin (5). Cu acts as a cofactor for several enzymatic activities and reactive proteins as tyrosinase, a protein responsible for pigmentation and lysyl oxidase which participates in the process of connective tissue development (6, 7). In the cellular respiration process, Cu is a cofactor for cytochrome-C oxidase, an enzyme responsible for the transfer of electrons at the end of the respiratory chain which ultimately generates energy for all tissues (8). Cu also participates in the process of free radical detoxification with the enzyme superoxide dismutase, as well as the mobilization of iron with ceruloplasmin (4, 8). The National Research Council (9) recommends 8 ppm supplementation of Cu at all stages of breeding. However, Cu has been widely used above the nutritional requirement for growth-promoting effects in poultry, and the use of high doses between 125 and 250 ppm has already been proven to be an efficient dosage range to improve performance in broilers (10, 11).

Zinc (Zn) is a micromineral that actively regulates the process of skeletal development, membrane protection, prostaglandin metabolism, and lipid metabolism (12). Zn is involved in the activity of more than 300 enzymes (13), and the metalloenzymes Zn- dependent are found in several tissues of the body where they perform functions related to the synthesis and degradation of carbohydrates, lipids and proteins. Furthermore, Zn is one of the constituents of the carbonic anhydrase metalloenzyme and acts on the acid-base balance of the organism (14). According to the National Research Council (9), the recommendation of zinc supplementation is 40 ppm for all stages of broiler breeding.

Coccidiosis damages intestinal epithelial morphology and reduces nutrient digestibility and performance of broiler chickens (15, 16). Moreover, the reduction of minearal absorption, especially in the duodenum, caused by coccidiosis might influence on bone development during grower phase. Different methods have been used as a form of prevention, including anticoccidials in the diet, vaccines, and supplementation of natural products (plant extracts and minerals). However, due to the resistance generated by the chemicals, new forms of prevention are necessary to be studied (3). We hypothesized that Cu and Zn can be effective agents to reduce detrimental effects of coccidiosis in broiler chickens. Thus, this study aimed to evaluate the effect of different levels of inorganic Cu and Zn on parameters of performance, bone quality, and antioxidant properties in broiler chickens during the challenge with *Eimeria* spp.

## Materials and Methods

### Bird Husbandry and Dietary Treatments

All the procedures performed in this study were approved by the Institutional Animal Care and Use Committee (IACUC) of University of Georgia (Athens, GA, USA). A total of 360 d-old male Cobb broiler chickens were housed in floor cages for 12 days at the Poultry Research Center. At 12 days of age, birds were placed in grower Petersime batteries and distributed in a completely randomized design with 10 birds per cage, six replicates per treatment, and six treatments. There were six corn-soybean meal-based dietary treatments: non-challenged control (NC), challenged control (CC), 100 ppm Cu (100 Cu), 150 ppm Cu (150 Cu), 80 ppm Zn (80 Zn), and 100 ppm Zn (100 Zn). The inorganic sources for the study were copper sulfate pentahydrate (CuSO_4_5H_2_O, Millipore Sigma, St. Louis, MO, USA) and zinc sulfate heptahydrate (ZnSO_4_7H_2_O, Millipore Sigma, St. Louis, MO, USA). Diets were isocaloric and isonitrogeous among the treatments and are represented in Table 1. Feed and water were provided *ad libitum*. Bird and feeder weights were recorded at d 12 and 20 post hatch for calculation of body weight (BW), body weight gain (BWG), feed intake (FI) and feed conversion ratio (FCR). On day 14, with exception of NC group, birds were challenged by oral gavage with a mix of *Eimeria maxima* (50,000 oocysts/bird), *Eimeria tenella* (50,000 oocysts/bird), and *Eimeria acervulina* (250,000 oocysts/bird). On day 20, eight birds randomly selected from each pen were euthanized to measure intestinal lesion scores according to a four-score scale (17).

**Table 1:** Ingredient (%) and composition of the experimental diets.

	**NC/CC^a^**	**100 Cu**	**150 Cu**	**80 Zn**	**100 Zn**
Corn, grain	60.01	60.01	60.01	60.01	60.01
Soybean meal −48%	34.15	34.15	34.15	34.15	34.15
Dical. phos.	1.58	1.58	1.58	1.58	1.58
Soybean oil	1.53	1.53	1.53	1.53	1.53
Limestone	1.17	1.17	1.17	1.17	1.17
Common salt	0.35	0.35	0.35	0.35	0.35
DL-met	0.29	0.29	0.29	0.29	0.29
Vitamin premix^b^	0.25	0.25	0.25	0.25	0.25
L-lysine-HCL	0.22	0.22	0.22	0.22	0.22
Mineral premix^c^	0.08	0.08	0.08	0.08	0.08
L-thr	0.07	0.07	0.07	0.07	0.07
Cr2O3	0.30	0.30	0.30	0.30	0.30
Copper sulfate, 25%	–	0.040	0.060	–	–
Zinc sulfate, 22.2%	–	–	–	0.036	0.045
**Nutrients**					
ME, kcal/kg	3,010	3,010	3,010	3,010	3,010
Crude protein	21.25	21.25	21.25	21.25	21.25
Lys%	1.32	1.32	1.32	1.32	1.32
Met%	0.63	0.63	0.63	0.63	0.63
TSAA%	0.98	0.98	0.98	0.98	0.98
Thr%	0.86	0.86	0.86	0.86	0.86
Ca%	0.90	0.90	0.90	0.90	0.90
avP %	0.45	0.45	0.45	0.45	0.45
**Analyzed composition**					
Cu, mg/kg	10.69	104.73	149.14	7.40	7.26
Zn, mg/kg	97.35	93.96	93.89	169.69	197.59

a *Non-challenged control (NC), Challenged control (CC), 100 ppm Cu (100 Cu), 150 ppm Cu (150 Cu), 80 ppm Zn (80 Zn), and 100 ppm Zn (100 Zn)*.

b *Provided per kg of DSM Vitamin premix: Vit. A 2,204,586 IU, Vit. D_3_ 200,000 ICU, Vit. E 2,000 IU, Vit. B12 2 mg, Biotin 20 mg, Menadione 200 mg, Thiamine 400 mg, Riboflavin 800 mg, d-Pantothenic Acid 2,000 mg, Vit. B6 400 mg, Niacin 8,000 mg, Folic Acid 100 mg, and Choline 34,720 mg*.

c *Provided per kg of Mineral premix: Ca 0.72 g, Mn 3.04 g, Zn 2.43 g, Mg 0.61 g, Fe 0.59 g, Cu 22.68 g, I 22.68 g, and Se 9.07 g*.

### Chemical Analysis

Cu and Zn were determined in all diets using the atomic absorption spectrometry method (FAAS - Flame Atomic Absorption Spectrometry), as described by Neves et al. (18).

### Intestinal Permeability

Fluorescein isothiocyanate dextran (FITC-d, 100 mg, MW 4000; Sigma-Aldrich, Canada) was used to determine intestinal permeability on 5 days post infection. One bird per cage from each treatment was gavaged with 2.2 mg FITC-d in 1 ml of phosphate buffered saline (PBS). After 2 h, blood samples were collected from wing veins and kept at room temperature for 3 h before being centrifuged (500 × g for 15 min) to obtain the serum. The serum was then diluted with PBS (1:1 PBS), and FITC-d in the serum was measured at an excitation wavelength of 485 nm and an emission wavelength of 528 nm using a multi-mode microplate fluorescence reader (SpectraMax M5, Molecular Devices, San Jose, CA, USA). Standards were spiked with FITC-d at 0, 0.2, 0.4, 0.6, 0.8, and 1.0 μg/mL to obtain a standard curve. Gut leakage for each bird was reported as μg of FITC-d /ml of serum (19).

### Oocyst Shedding

At the end of the experiment, samples of fresh excreta from each cage was collected to calculate oocyst shedding. The number of oocysts was determined using a McMaster counting chamber (McMaster Egg Counting Chamber, Vetlab Supply, Palmetto Bay, FL, USA) according to Hodgson (20), and the results were presented as per gram of excreta. In brief, total excreta from each cage were collected from 5 to 6 days post infection and mixed thoroughly to ensure uniformity. Additionally, a 5 g sample was transferred to a glass beaker and suspended with 50 mL of saturated sodium chloride solution. The suspension was mixed thoroughly and then left for 5 min to allow for the oocysts to float up before being used for calcuations. All oocysts under the grid of each chamber in the McMaster were counted.

### Antioxidant System

On d 20, serum and liver samples from one bird/cage were collected, and the liver was rinsed in ice-cold PBS and flash-frozen in liquid nitrogen. Within 24 h of harvest, tissues were homogenized in PBS containing 10 mM diethylenetriaminepentaacetic acid (DTPA) (Millipore Sigma, St. Louis, MO, USA), immediately acidified with 10% perchloric acid with 1 mM DTPA (Millipore Sigma, St. Louis, MO, USA), and homogenized for 25 s. Supernatants were filtered and flash-frozen in liquid nitrogen and stored at −80°C until further analysis. Concentrations of glutathione (GSH) and glutathione disulfide (GSSG) were measured by high performance liquid chromatography (HPLC; Dionex UltiMate 3000, Thermo Scientific, Waltham, MA, USA) coupled with electrochemical detection. The conditioning cell was set at +500 mV, and analytical cell was set at +1,475 mV with a cleaning potential of +1,900 mV for 30 s between samples. The mobile phase consisted of 3.0% acetonitrile, 0.1% pentafluoropropionic acid, and 0.02% ammonium hydroxide. The flow rate was maintained at 0.22 mL/min, and injection volumes were set at 5.0 μL. Peaks were quantified using external GSH and GSSG standards and the Chromeleon Chromatography Data System Software (Dionex Version 7.2, Thermo Fishier Scientific, Waltham, MA, USA). Total glutathione was calculated by the formula GSH + 2GSSG, and glutathione redox status was assessed by the ratio GSH/GSSG. Concentrations of GSH and GSSG were standardized to total protein, which was quantified by Pierce BCA Protein Assay (Thermo Fisher Scientific, Waltham, MA, USA).

The serum was collected by puncturing brachial veins, and superoxide dismutase activity was evaluated in the serum. The analysis was performed using commercial Superoxide Dismutase Assay Kits (Cayman Chemical, Ann Arbor, MI, USA) at 440 nm using a plate reader VICTOR (1420 Multilabel Counter, PerkinElmer, Waltham, MA, USA).

### Bone Quality

One bird from each experimental cage was euthanatized at d 20 by cervical dislocation for collecting the right tibia, which were immediately frozen at −20°C for further analysis.

In order to obtain the Seedor index, legs were thawed, and the adherent tissues were removed with a scalpel. The Seedor index is the relationship between length and weight of bone, and this relation is indicative of bone density (21). In this way, the *in natura* bones were measured at their longest length with a pachymeter, and their weight was obtained with a semi-analytical balance (Analytical balance ABT, Kern, Germany) with an accuracy of 0.001 gram. Bones were dried for 24 h at 105°C in a forced circulation oven (DESPATCH Forced Draft Ovens, Durham Geo-Enterprises, Inc, Stone Mountain, GA, USA), defatted in hexane using a Soxhlet extractor for 8 h and then taken back to the oven at 105°C for 12 h. After ashing bones for 4 h at 600°C, bone ash was determined based on defatted dry matter and expressed as ash percentage (22). Cu, Zn, calcium (Ca), magnesium (Mg), manganese (Mn) and phosphorus (P) levels in the bones were measured in the same bones used for Seedor index.

The mineral content was determined from bone ash, and Ca, Cu, Mn, Mg, and Zn contents were obtained by an atomic absorption spectrophotometer as described by Neves et al. (18), and P content by the spectrophotometric method of vanadomolybdic phosphoric acid according to Moraes et al. (23).

### Statistical Analysis

The data were analyzed by the PROC GLM of SAS (SAS Institute Inc., Cary, NC), and significant means were compared by Duncan's multiple-range test. Significance was declared when *P* ≤ 0.05.

## Results

### Growth Performance

The *Eimeria* spp. challenge reduced growth performance of broilers (Table 2), however the trace mineral supplementation did not significantly improve body weight, body weight gain, feed intake and feed conversion ratio. Birds in treatment 150 Cu had significantly lower feed intake compared to birds in treatment NC or 100 Cu, whereas there was no difference in feed conversion ratio between NC and 150 Cu.

**Table 2:** Growth performance of broilers challenged with *Eimeria* spp. and fed different levels of inorganic minerals (Cu and Zn) at 12–20 days of age, ^1^^,^^2^.

	**BW (kg)**	**BWG (kg)**	**FI (kg)**	**FCR (kg/kg)**
NC	0.784^a^	0.484^a^	0.759^a^	1.583^b^
CC	0.658^b^	0.357^b^	0.668^bc^	1.876^a^
100 Cu	0.668^b^	0.368^b^	0.669^b^	1.820^a^
150 Cu	0.651^b^	0.351^b^	0.621^c^	1.778^ab^
80 Zn	0.636^b^	0.335^b^	0.651^bc^	1.965^a^
100 Zn	0.657^b^	0.356^b^	0.656^bc^	1.844^a^
***P-*****Value**	<0.0001	<0.0001	<0.0001	0.015
**SE**	9.974	9.971	9.341	0.033

1 *Non-challenged control (NC), Challenged control (CC), 100 ppm Cu (100 Cu), 150 ppm Cu (150 Cu), 80 ppm Zn (80 Zn), and 100 ppm Zn (100 Zn); SE: standard error*.

2 *BW: body weight; BWG: body weight gain; FI: feed intake; FCR: feed conversion ratio (FI/BWG)*.

a, b, c *mean within a column with different superscripts are significantly different (P < 0.05). Values of mean represent 6 replicated pens per treatment*.

### Gut Health and Oocyst Shedding

The results from intestinal permeability (Table 3) showed the concentration of FITC-d (μg/ml) in the serum was reduced (*P* = 0.0004) with the inclusion of 100 ppm Zn in the diet and reached a similar level to NC group. The 80 Zn and 100 Zn groups had significantly lower FITC-d level than 100 Cu group.

**Table 3:** FITC-d concentration in serum of broilers challenged with *Eimeria* spp. and fed different levels of inorganic minerals (Cu and Zn), ^1^.

	**FITC-d μg/ml**
NC	0.0071^c^
CC	0.0936^ab^
100 Cu	0.1272^a^
150 Cu	0.1113^ab^
80 Zn	0.0580^bc^
100 Zn	0.0375^c^
***P*****-value**	0.0004
**SE**	0.00995

1 *Non-challenged control (NC), Challenged control (CC), 100 ppm Cu (100 Cu), 150 ppm Cu (150 Cu), 80 ppm Zn (80 Zn), and 100 ppm Zn (100 Zn); SE: standard error*.

a, b, c *mean within a column with different superscripts are significantly different (P < 0.05). Values of mean represent 6 replicated pens per treatment*.

In the present study, lesion score was significant higher in CC group compared to NC group. A lower lesion score in the duodenum of broilers fed 150 ppm Cu compared to CC group was observed (*P* < 0.0001; Table 4). However, there were no differences in lesion scores of jejunum and ileum among the challenged groups. In ceca, 80 or 100 Zn treatments showed significantly lower lesion score than 100 Cu.

**Table 4:** Intestinal lesion score at 20 days of age of broilers challenged with *Eimeria* spp. and fed different levels of inorganic minerals (Cu and Zn), ^1^.

	**Duodenum**	**Jejunum and ileum**	**Ceca**
NC	0^c^	0^b^	0^c^
CC	3.58^a^	3.15^a^	2.34^ab^
100 Cu	3.21^ab^	3.29^a^	2.52^a^
150 Cu	2.80^b^	2.95^a^	2.33^ab^
80 Zn	3.29^ab^	2.95^a^	2.04^b^
100 Zn	3.15^ab^	3.00^a^	2.11^b^
***P*****-Value**	<0.0001	<0.0001	<0.0001
**SE**	0.2155	0.1995	0.1535

1 *Non-challenged control (NC), Challenged control (CC), 100 ppm Cu (100 Cu), 150 ppm Cu (150 Cu), 80 ppm Zn (80 Zn), and 100 ppm Zn (100 Zn); SE: standard error*.

a, b, c *mean within a column with different superscripts are significantly different (P < 0.05). Values of mean represent 6 replicated pens per treatment*.

For the oocyst shedding results, 150 Cu, 80 Zn, and 100 Zn treatments significantly increased *Eimeria maxima* shedding compared to CC group. The inclusion of 80 and 100 ppm inorganic Zn treatments (80 and 100 Zn) signficantly reduced the *Eimeria acervulina shedding* and total shedding counts compared to CC group (Table 5).

**Table 5:** Oocyst shedding (oocyst/gram) at day 6 postinfection of broilers challenged with *Eimeria* spp. and fed different levels of inorganic minerals (Cu and Zn), ^1^.

	***E. maxima***	***E. tenella***	***E. acervulina***	**Total**
CC	30,237^c^	89,378	1,236,173^a^	1,355,789^a^
100 Cu	63,587^bc^	77,817	1,494,525^a^	1,635,929^a^
150 Cu	91,601^ab^	61,364	1,295,759^a^	1,448,724^a^
80 Zn	121,839^a^	58,251	782,169^b^	962,259^b^
100 Zn	125,174^a^	59,474	724,473^b^	909,121^b^
***P*****-value**	0.0011	0.1429	0.0003	0.0021
**SE**	9,573.65	4,738.91	74,739.99	75,774.97

1 *Non-challenged control (NC), Challenged control (CC), 100 ppm Cu (100 Cu), 150 ppm Cu (150 Cu), 80 ppm Zn (80 Zn), and 100 ppm Zn (100 Zn); SE: standard error*.

a, b, c *mean within a column with different superscripts are significantly different (P < 0.05). Values of mean represent 6 replicated pens per treatment*.

### Glutathione and Superoxide Dismutase

The CC group increased GSH and GSH + 2 GSSG and reduced GSH/GSSG as compared to NC group (Table 6), indicating that there was an upregulation in both GSH and GSSG production but GSH was rapidly oxidized to GSSG as a result of oxidative stress caused by *Eimeria* challenge. Birds in 100 Cu and 150 Cu groups reduced GSH and GSH + 2GSSG levels back to the levels similar to those of NC group, whereas birds in 80 Zn and 100 Zn had lower production of GSH, GSSG, GSH + 2GSSG but higher GSH/GSSG ratio than CC group, suggesting that Cu and Zn supplementation improved antioxidant capability against oxidative stress caused by *Eimeria* infection. However, the activity of the superoxide dismutase was not affected by *Eimeria* spp. challenge, even when birds received diets supplemented with Cu and Zn (Table 6).

**Table 6:** The amount of glutathione (GSH), glutathione disulfide (GSSG), total glutathione (GSH + 2GSSG), glutathione redox status (GSH/GSSG), and the activity of superoxide dismutase of broilers challenged with *Eimeria* spp. and fed different levels of inorganic minerals (Cu and Zn), ^1^.

	**GSH (nM/mg)**	**GSSG (nM/mg)**	**GSH + 2GSSG (nM/mg)**	**GSH/GSSG**	**Superoxide dismutase (U/ml)**
NC	25.59^b^	0.10^b^	25.79^b^	315.67^a^	12.89
CC	34.67^a^	0.37^ab^	35.42^a^	96.82^cd^	12.53
100 Cu	24.27^b^	0.75^ab^	25.77^b^	71.81^d^	12.19
150 Cu	18.66^b^	1.07^a^	20.82^b^	61.46^d^	12.61
80 Zn	19.73^b^	0.37^ab^	20.48^b^	193.30^bc^	12.55
100 Zn	23.88^b^	0.11^b^	24.11^b^	248.34^ab^	12.20
***P*****-value**	0.0092	0.0435	0.014	<0.0001	0.8189
**SE**	1.41	0.11	1.39	21.53	0.1597

1 *Non-challenged control (NC), Challenged control (CC), 100 ppm Cu (100 Cu), 150 ppm Cu (150 Cu), 80 ppm Zn (80 Zn), and 100 ppm Zn (100 Zn); SE: standard error*.

a, b, c, d *mean within a column with different superscripts are significantly different (P < 0.05). Values of mean represent 6 replicated pens per treatment*.

### Bone Quality

The parameters of bone quality and mineralization in *Eimeria* challenged fed Cu and Zn supplemented diets are present in Table 7. Birds in 100 Cu group showed higher tibia ash (%) as compared to other treatments. The Seedor index and mineral concentrations were not influenced by any treatments (Table 7).

**Table 7:** Bone quality of broilers challenged with *Eimeria* spp. and fed different levels of inorganic minerals (Cu and Zn), ^1^^,^^2^.

	**Seedor index**	**Ash (%)**	**Cu (ppm)**	**Zn (ppm)**	**Ca (g/g bone ash)**	**Mg (g/g bone ash)**	**Mn (g/g bone ash)**	**P (g/g bone ash)**
NC	0.086	39.62^b^	<10.0	389.21	0.4428	0.0105	<10.0	0.213
CC	0.080	39.10^b^	<10.0	367.67	0.3926	0.0092	<10.0	0.1884
100 Cu	0.082	51.49^a^	<10.0	389.01	0.3288	0.007167	<10.0	0.1568
150 Cu	0.084	39.22^b^	<10.0	369.39	0.4027	0.00933	<10.0	0.1935
80 Zn	0.079	40.18^b^	<10.0	389.19	0.3740	0.00883	<10.0	0.1800
100 Zn	0.083	38.53^b^	<10.0	379.51	0.3967	0.009167	<10.0	0.1898
***P*****-value**	0.3891	<0.0001	1	0.9083	0.1316	0.0163	1	0.1019
**SE**	0.0011	1.041	0	7.033	0.012044	0.000283	0	0.005714

1 *Non-challenged control (NC), Challenged control (CC), 100 ppm Cu (100 Cu), 150 ppm Cu (150 Cu), 80 ppm Zn (80 Zn), and 100 ppm Zn (100 Zn); SE: standard error*.

2 *Ash: bone ash weight/bone fat free dry weight %*.

a, b *mean within a column with different superscripts are significantly different (P < 0.05). Values of means represent 6 replicated pens per treatment*.

## Discussion

The different levels of Cu and Zn supplementation did not improve growth performance of broilers during *Eimeria* infection. Previous studies have reported that high Cu supplementation (125–250 ppm) in diets of broilers improved growth performance, because the trace mineral has an antimicrobial effect (10, 11). On the other hand, the poor performance observed can be related to the period when the birds received the trace mineral supplementation (12–20 days) that probably was not sufficient to reduce the deleterious effects caused by the infection. Similar results were observed by (24, 25) reporting that Zn supplementation was not effective to improve growth performance during *Eimeria acervulina* or *Eimeria tenella* infection. According to Johnson and Reid (17), each species of *Eimeria* invade a specific section of the intestine; for instance, *Eimeira acervulina* causes damage in the duodenum, which is also the site for greater absorption of Zn, *Eimeria maxima* causes infection in the mid-intestinal region, and *Eimeria tenella* affects the ceca (26). In study of (27), they reported that after oocysts infection in the gastrointestinal tract, they sporulate and enter in enterocytes, and *Eimeria* sporozoites may reduce the absorption of nutrients within the enterocytes. In addition, *Eimeria* cells compete for energy and nutrients within the enterocytes.

The *Eimeria* spp. mainly compromise the gut heath, leading to a reduction of nutrients absorption, which is in agreement with the results in the present study showing that birds from CC group showed increased intestinal permeability, high incidence of lesion score (duodenum, jejunum & ileum and ceca) and higher *E. acervulina* oocyst shedding.

In the current study, Zn supplementation reduced the absorption of FITC-d in the gut, indicating that Zn minimized intestinal damage by *Eimeria* spp.; however, no positive effects on intestinal permeability in Cu treatments were observed. Supplementation of high doses of Zn can be important due to its critical roles involved in gut health as an antimicrobial, antioxidant and other immune properties (8). Although the growth performance was not improved by Zn supplementation, at intestinal level, this supplementation was efficacious to improve intestinal integrity and reduce gut damage caused by *Eimeria* spp. A study demonstrated a role of Zn during a gastrointestinal disease and concluded that Zn supplementation can reduce diarrhea because this trace mineral can positively affect multiple aspects of gastrointestinal mucosa and enhance the intestinal barriers (28). The intestinal epithelial barrier is formed by intercellular junctional complexes, including tight junctions, adherens junctions and desmosomes. The tight junctions are formed by a set of proteins including claudin, occludin, junctional adhesion molecule, and zona occludens, which are responsible for the permeability of the paracellular pathway (29). The increased permeability is the result of some injury to the intestinal barrier and may allow for the passage of antigenic agents, with consequent reduction in nutrient absorption. A study evaluated the permeability by measuring lactulose and mannitol in the urine of pigs receiving diets supplemented with inorganic Zn (30) and showed a lower presence of these compounds in the urine of Zn supplemented pigs compared to the control group. Lactulose crosses the intestinal barrier via intercellular tight junctions of the epithelium of the crypts, while mannitol crosses through the transcellular epithelium of the villus.

Cu has an antibacterial property when pharmacological level is used in diets of broilers; it improves growth performance as a consequence of a reduction of pathogenic bacteria and better absorption of nutrients (10, 11). We found that 150 ppm Cu supplementation significantly reduced lesion scores in the duodenum of broilers challenged with mixed *Eimeria* spp. The current result is in agreement with a study (31) reporting that Cu supplementation minimized intestinal damage in broilers challenged with *Eimeria tenella*. This suggests that Cu supplementation could be one of the nutritional strategies to reduce intestinal damage and improve gut health in broilers.

As mentioned previously, each *Eimeria* invades and damages a specific region of the intestine. In the current study, Zn supplementation significantly reduced oocyst shedding of *Eimeria acervulina*, which affects the duodenum, as well as minimized serum FITC-d level, indicating that Zn could be a good candidate mineral to reduce detrimental effects of *Eimeria* infection and maintain intestinal integrity and health in broilers.

Manifestation of oxidative stress increased production of free radicals which causes damage not only on pathogens, but also host' cells (32, 33). Key enzymes in antioxidant defense systems, including glutathione (reductase and peroxidase), catalase and superoxide dismutase, are necessary to reduce oxidative damages (32). Among them, the glutathione peroxidase is involved in regulating the production of glutathione (GSH) and glutathione disulfide (GSSG) that are indicative of the oxidative stress status. In the current study, *Eimeria* infection significantly increased GSH, GSSG, and GSH + GSSG but reduced GSH/GSSG ratio. Although increased levels of GSH, a reduced form of glutathione, can be an indicator of antioxidant capacity, a high production of GSH can be induced by a high amount of hydrogen peroxide as a result of oxidative stress (34). Thus, GSH/GSSG is a better indicator for the status of the body antioxidant capacity. The current study showed that Zn supplementation increased GSH/GSSG, indicating that Zn supplementation improves antioxidant capacity and reduce oxidative stress caused by *Eimeria* infection in broilers.

Since trace minerals such as Cu and Zn act as co-factors for the enzyme superoxide dismutase (35), Cu and Zn supplementation could increase Cu and Zn availability for this enzyme that neutralizes free radicals to reduce oxidative stress. A study reported greater activity of superoxide dismutase in broilers fed 40 and 60 ppm of organic Zn at 42 days of age, challenged with *Eimeria tenella* due to an antioxidant role of Zn (25). However, we did not observe positive effects of Zn on the activity of this enzyme, suggesting that this may be related to the species of *Eimeria* used and possibly to the life cycle of the parasites, as reported by Georgieva et al. (36) who found lower superoxide dismutase activity in the serum of broilers supplemented with Zn after 8 days post infection with *Eimeria acervulina*.

According to the current results, 100 ppm Cu supplementation in diets of broilers in challenge led to an increase of tibia ash when compared to other treatments. In this way, seems like the copper absorption occurred normally and benefited the bone ash, but was not efficacious to improve the growth performance. On contrary (37, 38), evaluated bone parameters of broilers challenged with *Eimeria maxima* and *Eimeria acervulina* 6 days post infection and observed that bone ash and mineralization were compromised in challenged group compared to the control group. The main factors that cause changes in bone formation are physical, nutritional, physiological, as well as the immune system (39, 40). Evaluated the response of challenged chickens with *Eimeria* and hypothesized that the release of tumor necrosis factor (TNF-α), IL-1 and IL-9 increased bone resorption, and therefore, challenged chickens had worse bone quality parameters (41).

As we know, the pharmacological levels of Cu or Zn supplementation acts on improving parameters of performance, antioxidant status and many other roles that these minerals are involved. During *Eimeria* challenge, high supplementation of Cu and Zn did not work as expect however, many factors can be related, for instance, mineral source and time of supplementation. More studies are need to clarify how is the best way to use Cu and Zn in diets of broilers during the *Eimeria* challenge.

## Conclusion

In summary, the inorganic Cu and Zn supplementation during the *Eimeria* challenge showed the positive effects on intestinal health, oocyst shedding and antioxidant properties. The supplementation of 150 ppm Cu reduced the duodenum lesion scores. The supplementation of Zn decreased the intestinal permeability, the ceca lesion score, the *Eimeria acervulina* and total oocyst shedding and showed a positive effect on antioxidant properties by increasing GSH/GSSG ratio. Both Cu and Zn showed the positive properties to alleviate detrimental effects of *Eimeria* challenge and can be potential mineral additives for broiler to combat the coccidiosis.

## Data Availability Statement

The datasets generated for this study are available on request to the corresponding author.

## Ethics Statement

The animal study was reviewed and approved by the Institutional Animal Care and Use Committee (IACUC) of University of Georgia.

## Author Contributions

AF provided the the oocysts of Eimeria spp for the study. SY and FC carried out the entire experiment. The glutathione analysis was performed by RP, RG and SC. The manuscript was written by TS, WK, CC, P-YT and JS.

## Conflict of Interest

The authors declare that the research was conducted in the absence of any commercial or financial relationships that could be construed as a potential conflict of interest.
